# Low risk pregnancies after a cesarean section: Determinants of trial of labor and its failure

**DOI:** 10.1371/journal.pone.0226894

**Published:** 2020-01-13

**Authors:** Sjur Lehmann, Elham Baghestan, Per E. Børdahl, Lorentz M. Irgens, Svein Rasmussen

**Affiliations:** 1 Department of Clinical Science, University of Bergen, Bergen, Norway; 2 Department of Obstetrics and Gynecology, Haukeland University Hospital, Bergen, Norway; 3 Department of Global Public Health and Primary Care, University of Bergen, Bergen, Norway; 4 Medical Birth Registry of Norway, Norwegian Institute of Public Health, Bergen, Norway; Oslo Universitetssykehus Ulleval, NORWAY

## Abstract

**Introduction:**

In pregnancies after a previous cesarean section (CS), a planned repeat CS delivery has been associated with excess risk of adverse outcome. However, also the alternative, a trial of labor after CS (TOLAC), has been associated with excess risks. A TOLAC failure, involving a non-planned CS, carries the highest risk of adverse outcome and a vaginal delivery the lowest. Thus, the decision regarding delivery mode is pivotal in clinical handling of these pregnancies. However, even with a high TOLAC rate, as seen in Norway, repeat CSs are regularly performed for no apparent medical reason. The objective of the present study was to assess to which extent demographic, socioeconomic, and health system factors are determinants of TOLAC and TOLAC failure in low risk pregnancies, and whether any effects observed changed with time.

**Materials and methods:**

The study group comprised 24 645 second deliveries (1989–2014) after a first delivery CS. Thus, none of the women had prior vaginal deliveries or more than one CS. Included pregnancies were low risk, cephalic, single, and had gestational age ≥ 37 weeks. Data were obtained from the Medical Birth Registry of Norway (MBRN). The exposure variables were (second delivery) maternal age, length of maternal education, maternal country of origin, size of the delivery unit, health region (South-East, West, Mid, North), and maternal county of residence. The outcomes were TOLAC and TOLAC failure, as rates (%), relative risk (RR) and relative risk adjusted (ARR). Changes in determinant effects over time were assessed by comparing rates in two periods, 1989–2002 vs 2003–2014, and including these periods in an interaction model.

**Results:**

The TOLAC rate was 74.9%, with a TOLAC failure rate of 16.2%, resulting in a vaginal birth rate of 62.8%. Low TOLAC rates were observed at high maternal age and in women from East Asia or Latin America. High TOLAC failure rates were observed at high maternal age, in women with less than 11 years of education, and in women of non-western origin. The effects of health system factors, i.e. delivery unit size and administrative region were considerable, on both TOLAC and TOLAC failure. The effects of several determinants changed significantly (*P* < 0.05) from 1989–2002 to 2003–2014: The association between non-TOLAC and maternal age > 39 years became weaker, the association between short education and TOLAC failure became stronger, and the association between TOLAC failure and small size of delivery unit became stronger.

**Conclusion:**

Low maternal age, high education, and western country of origin were associated with high TOLAC rates, and low TOLAC failure rates. Maternity unit characteristics (size and region) contributed with effects on the same level as individual determinants studied. Temporal changes were observed in determinant effects.

## Introduction

In pregnancies after a previous cesarean section (CS), a planned repeat CS delivery has been associated with excess risk of adverse outcome, e.g. maternal death, prolonged hospitalization, and subsequent pregnancy complications. However, excess risks have also been reported in trial of labor after CS (TOLAC), e.g. uterine rupture, bleeding with needed transfusion, infection, and fetal compromise. Since the risk of adverse outcome is lowest in successful TOLAC and highest in TOLAC failure decisions regarding mode of delivery are pivotal in optimal clinical handling of these pregnancies [1].

Several medical conditions, i.e. obesity and macrosomia, have been associated with TOLAC failure and might warrant a planned repeat CS [2]. However, in uncomplicated cases evidence supports promotion of TOLAC as an important strategy to avoid unnecessary cesareans [3]. Still, declining TOLAC rates have been reported in low risk pregnancies [2] and even with a high TOLAC rate, as seen in Norway, repeat CSs are regularly performed for no apparent medical reason [2–4].

Various non-medical factors, e.g. ethnic and socioeconomic background, have been associated with TOLAC and TOLAC failure in previous research [3, 5, 6]. Some of these, i.e. maternal age and ethnicity, have been included in clinical nomograms intended for estimation of individual TOLAC failure probability prior to onset of delivery [5, 6]. However, effects could be time- and place-dependent, possibly limiting transferability. Furthermore, the characteristics of the health system and delivery unit might be relevant [1, 3].

Thus, the objective of the present study was to assess to which extent demographic, socioeconomic, and delivery unit characteristics are determinants of TOLAC and TOLAC failure in low risk pregnancies in Norway. Additionally, we wanted to assess temporal changes in the effects of such determinants during the observation period.

## Materials and methods

Since 1967, based on compulsory notification, the Medical Birth Registry of Norway (MBRN) has received clinical data on all births in the country, including medical conditions, complications, interventions, and outcome of mother and child. Shortly after delivery, a notification form is filled in by midwives and doctors at the delivery unit, and completed at discharge with additional data on post-delivery events, including a mandatory pediatric examination of the newborn [7]. Data necessary for identification of TOLAC deliveries in the MBRN were available from 1989 through 2014. By the national identification number of each woman, we linked MBRN data on the first and second birth and data on maternal education and country of origin [8].

By this method, 68 878 second deliveries after a first delivery CS were identified, of which 57 109 pregnancies were cephalic, single, and with a gestational age ≥ 37 weeks. Thus, none of the women included had any prior vaginal deliveries or more than one CS delivery. A low risk study group of 24 645 pregnancies was established by excluding women with recorded medical risk factors, i.e. second pregnancy record of assisted reproduction, diabetes mellitus type 1 or 2, gestational diabetes, cardiac disease, asthma, kidney failure, thyroid disease, epilepsy, hypertensive disease, rheumatoid arthritis, psychiatric diagnoses, small for gestation age (SGA), macrosomia, placenta previa, pregnancy induced hypertensive disorders, or major malformations [9]. We also excluded women with a first delivery record of stillbirth, cephalopelvic disproportion, or prolonged labor.

The exposure variables, i.e. the potential determinants, were second delivery characteristics available from the MBRN or Statistics Norway: maternal age (<25, 25–29, 30–34, 35–39, >39), length of maternal education (<11 years, 11–14 years, >14 years), maternal country of birth [8] size of the delivery unit (<500, 500–1499, 1500–2999, and ≥3000 deliveries per year), health region of the delivery unit (South-East, West, Mid, North), and mothers county of residence at the time of delivery.

**Fig 1:**
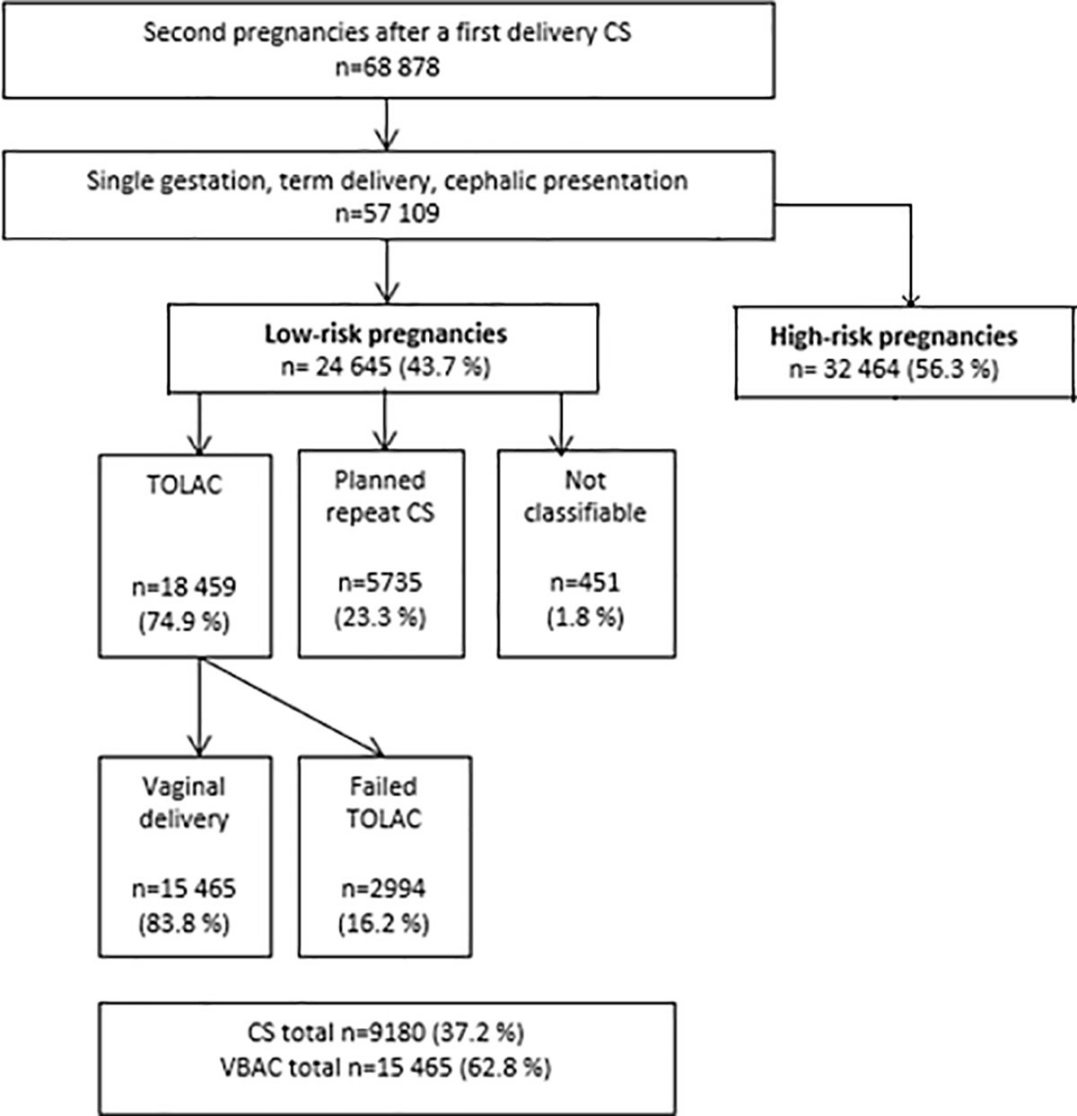
Study population, distribution of trial of labor after cesarean section (TOLAC) and planned repeat CS. Within the TOLAC group: Distribution of successful TOLAC (vaginal deliveries) and failed TOLAC, Norway 1989–2004.

The outcome variables were TOLAC and TOLAC failure. To identify TOLAC deliveries by MBRN data, a previously validated method was employed [10, 11]: The TOLAC group comprised all vaginal deliveries and acute or unspecified CS deliveries with a record of induced or augmented labor, labor complications, or delivery beyond 279 days gestational age. The non-TOLAC group comprised any CS recorded as planned, elective, or performed before onset of labor. A TOLAC failure case was defined as a TOLAC case with a CS delivery. By this method, 451 deliveries were unclassifiable (1.8% of the low risk group) (Fig 1). These deliveries comprised acute or unspecified CSs before 280 days of gestation, after a spontaneous onset of labor, combined with missing data on plan of delivery and no record of augmented labor or labor complications.

Excluding unclassified deliveries, the relative risk (RR) of TOLAC and TOLAC failure was calculated for each potential determinant, crude and adjusted (ARR) in a log-binomial model including year of birth, maternal age, maternal education, maternal country of origin, and size of delivery unit, with a 95% CI. Temporal changes in the effect of the potential determinants were explored by comparing determinant specific rate in the first and last part of the observation period (1989–2002 vs 2003–2014). Significance was tested by calculating RR and ARR, including an interaction term in the model, between a time period variable and each determinant studied.

IBM SPSS software version 20.0 was used for the statistical analyses. The Regional Committee for Medical and Health Research Ethics approved the study (REK Vest cases no 2012/1466 and 2015/1728).

## Results

**Fig 2:**
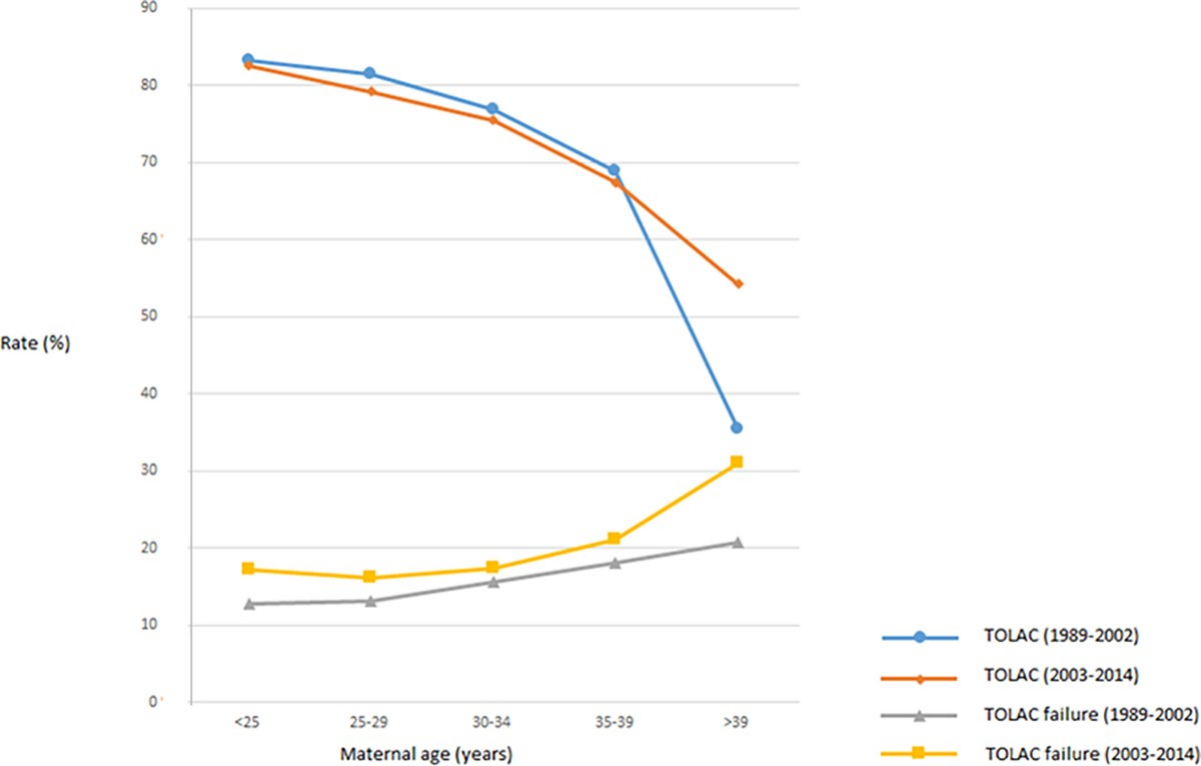
Trial of labor after cesarean section (TOLAC) and TOLAC failure rate (%) by maternal age in two time-intervals, low risk pregnancies, Norway 1989–2014.

The TOLAC rate in the 24 645 low risk deliveries studied was 74.9%. With a TOLAC failure rate of 16.2%, this resulted in a VBAC rate of 62.8% (Fig 1). TOLAC was associated with low maternal age (Table 1, Fig 2). Furthermore, in women with age 40 +, the TOLAC rate increased from 1989–2002 to 2003–2014 (Fig 2) (*P*<0.05). TOLAC failure was associated with high maternal age (Table 1, Fig 2). In women with age 40 +, the TOLAC failure rate increased from 1989–2002 to 2003–2014, however not significantly.

**Table 1:** Trial of labor after cesarean section (TOLAC) and TOLAC failure in low risk pregnancies by maternal characteristics and size of delivery unit, Norway 1989–2014. Rate, relative risk (RR) and RR adjusted (ARR) for maternal age, origin, education, and delivery unit size.

			TOLAC									Failed TOLAC						
Determinant		N	n	%	RR	95% CI		ARR	95% CI		n	%	RR	95% CI		ARR	95% CI	
Maternal age (years)																		
	<25	2532	2052	81.0	Reference = 1						297	14.5	Reference = 1					
	25–29	8278	6554	79.2	0.97	0.95	0.99	0.96	0.94	0.98	941	14.4	0.99	0.88	1.12	1.04	0.92	1.18
	30–34	9383	7019	74.8	0.92	0.90	0.94	0.89	0.88	0.91	1166	16.6	1.15	1.02	1.29	1.24	1.10	1.41
	35–39	3761	2512	66.8	0.82	0.80	0.84	0.80	0.78	0.82	499	19.9	1.37	1.20	1.57	1.49	1.30	1.70
	>39	691	322	46.6	0.57	0.53	0.62	0.57	0.53	0.62	91	28.3	1.95	1.59	2.39	2.08	1.69	2.56
Maternal education (years)																		
	<11	11713	8691	74.2	Reference = 1							1477	17.0	Reference = 1					
	11–14	9882	7473	75.6	1.02	1.00	1.03	1.04	1.03	1.06	1140	15.3	0.90	0.84	0.96	0.93	0.86	1.00
	>14	2704	2020	74.7	1.00	0.97	1.02	1.04	1.02	1.07	327	16.2	0.95	0.85	1.06	0.95	0.85	1.07
	Missing	346	275	79.5							50	18.2						
Maternal origin (country)																		
	Norway	20245	15245	75.3	Reference = 1							2322	15.2	Reference = 1					
	Europe	1247	931	74.7	0.99	0.96	1.02	1.01	0.98	1.04	146	15.7	1.03	0.88	1.20	0.96	0.82	1.12
	North America	130	101	77.7	1.03	0.94	1.13	1.03	0.95	1.13	13	12.9	0.85	0.51	1.41	0.85	0.51	1.41
	Latin America and the Caribbean	187	112	59.9	0.80	0.71	0.90	0.83	0.74	0.93	31	27.7	1.82	1.34	2.46	1.67	1.24	2.26
	West Asia, South Asia, North Africa	837	634	75.7	1.02	0.98	1.06	1.00	0.97	1.04	136	21.5	1.41	1.21	1.64	1.43	1.22	1.67
	Africa south of the Sahara	511	410	80.2	1.07	1.03	1.12	1.07	1.03	1.11	153	37.3	2.45	2.15	2.79	2.30	2.01	2.64
	East Asia, South-East Asia	468	293	62.6	0.85	0.79	0.91	0.87	0.81	0.93	72	24.6	1.61	1.32	1.98	1.47	1.20	1.80
	Oceania	9	8	88.9							1	12.5						
	Missing	1011	725	71.7							121	16.5						
Size of unit (deliveries/year)																		
	≥3000	8687	6677	76.9	Reference = 1							1030	15.4	Reference = 1					
	1500–2999	7332	5356	73.0	0.96	0.94	0.97	0.96	0.94	0.97	915	17.1	1.11	1.02	1.20	1.16	1.07	1.26
	500–1499	5937	4445	74.9	0.98	0.96	1.00	0.96	0.95	0.98	731	16.4	1.07	0.98	1.16	1.20	1.10	1.32
	<500	2650	1942	73.3	0.96	0.93	0.98	0.95	0.92	0.97	318	16.4	1.06	0.95	1.19	1.22	1.09	1.37
	Missing/not hospital	39	39	100							0	0						
**Total**		**24645**	**18459**	**74.9**							**2994**	**16.2**						

**Fig 3:**
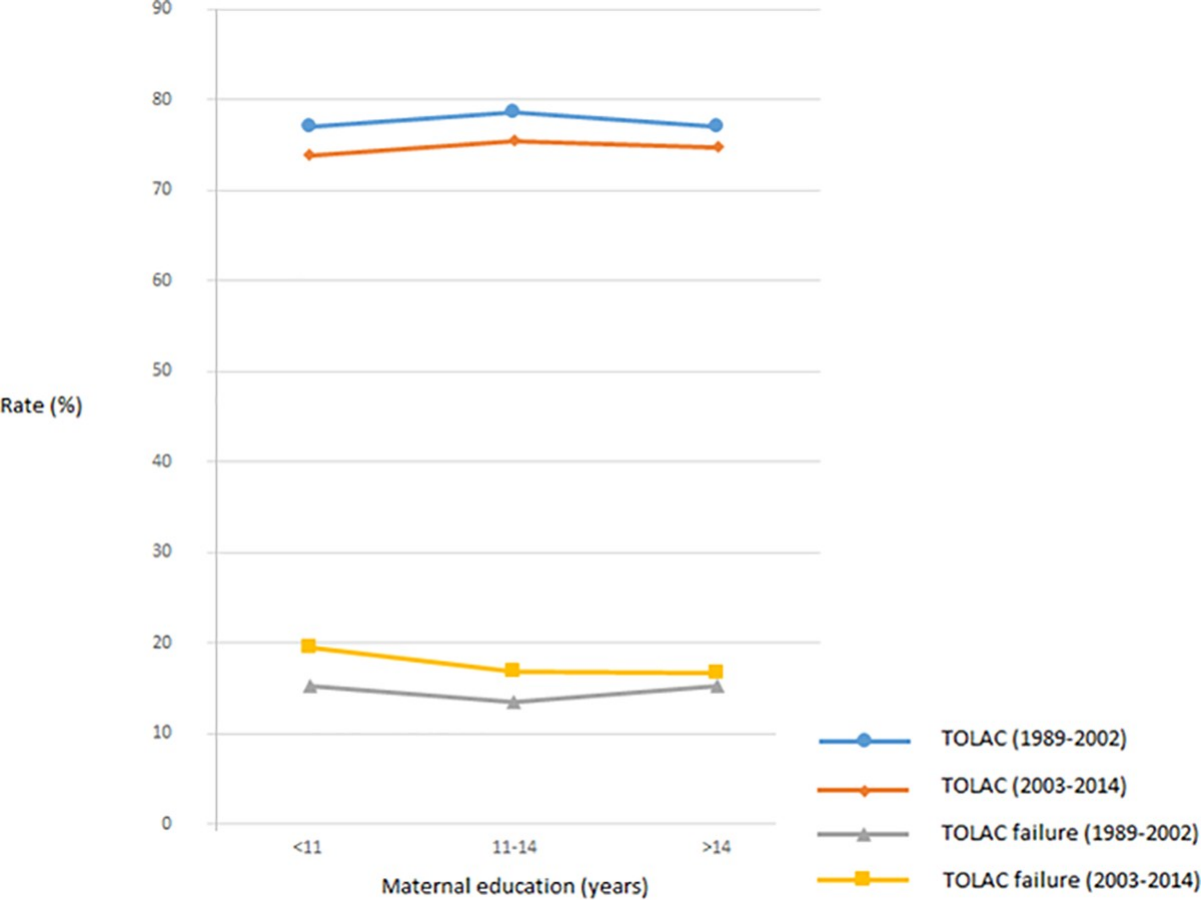
Trial of labor after cesarean section (TOLAC) and TOLAC failure rate (%) by maternal education in two time-intervals, low risk pregnancies, Norway 1989–2014.

In crude analyses, no association between TOLAC and the length of maternal education was observed (Table 1, Fig 3). However, after adjustment, TOLAC was associated with an education of more than 11 years. The TOLAC failure risk was higher in women with an education of less than 11 years (17.0%) compared to those with an education length 11 to 14 years (15.3%) and 14 + years (16.2%) (Table 1). In the second period, the association between short education and TOLAC failure was stronger (Fig 3).

In women from South-East Asia and Latin America/the Caribbean (Table 1), low TOLAC rates were observed, 62.2% and 59.9% respectively, and high TOLAC rates were observed in women from Africa south of the Sahara (80.2%). Excess TOLAC failure risk was present in all women of non-western origin (Table 1).

**Fig 4:**
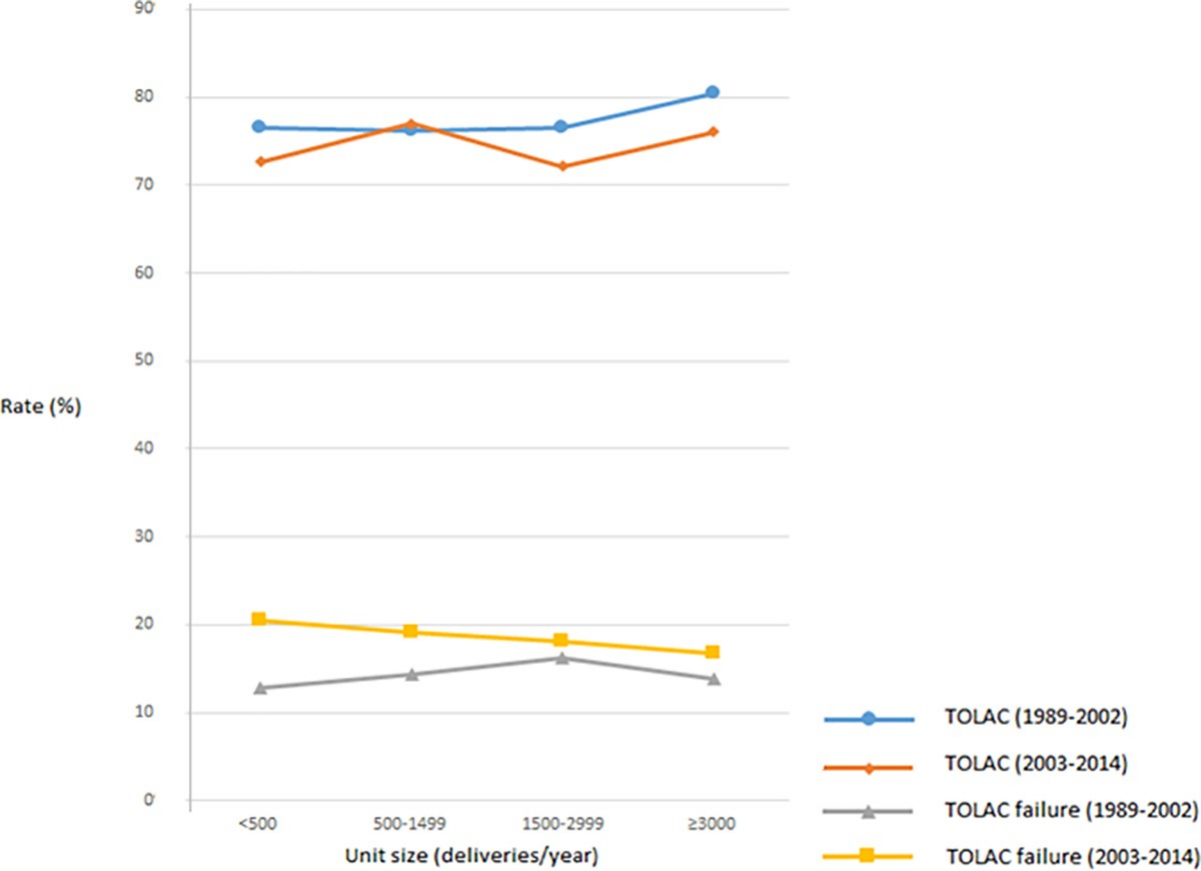
Trial of labor after cesarean section (TOLAC) and TOLAC failure rate (%) by delivery unit size (deliveries/year) in two time-intervals, low risk pregnancies, Norway 1989–2014.

In units with > 3000 yearly deliveries (Table 1). TOLAC rates were higher (76.9%) than in smaller units (1500–2999, 73.0%; 500–1499, 74.9%, < 500, 73.3%). In units with > 3000 yearly deliveries (Table 1) TOLAC failure rates were lower (15.4%) than in smaller units (1500–2999, 17.1%; 500–1499, 16.4%, < 500, 16.4%) (Table 1). In the second half of the observation period, a stronger association was seen between small unit size and TOLAC failure (Fig 4).

**Fig 5:**
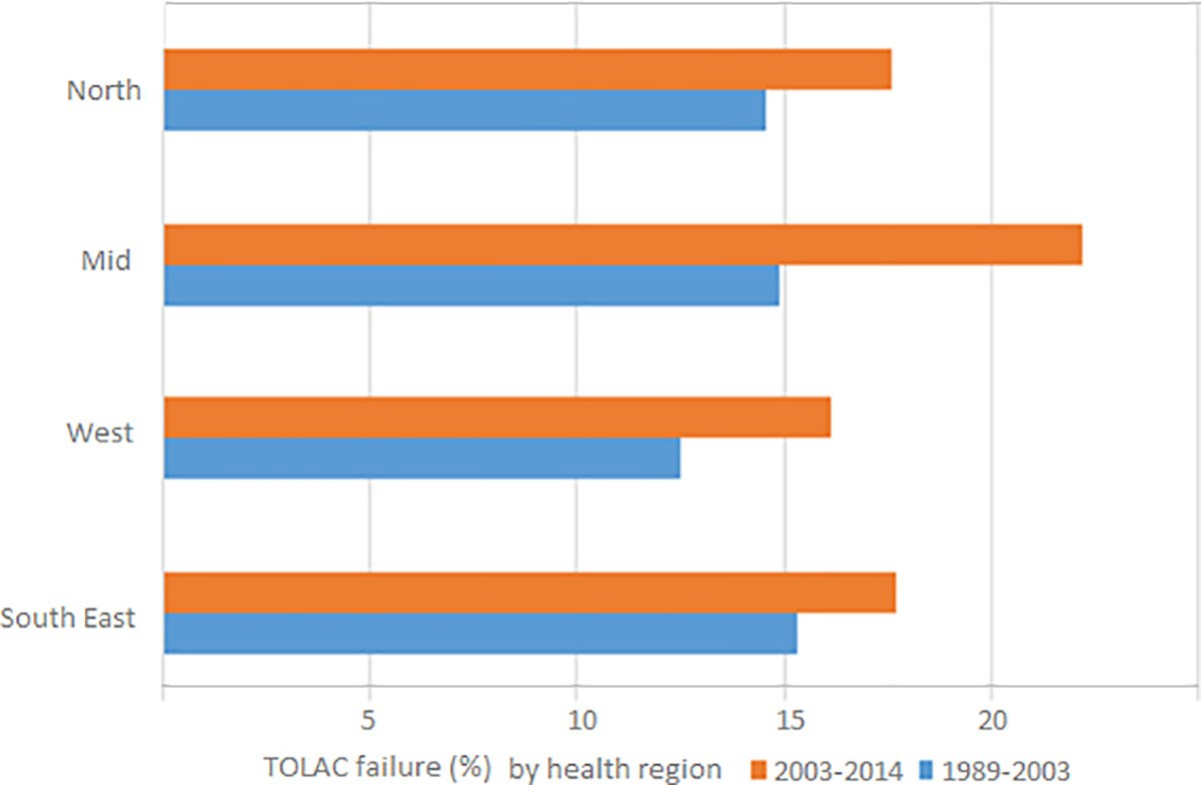
Trial of labor after cesarean section (TOLAC) and TOLAC failure rate (%) by health region (North, Mid, West, South East) in two time-intervals, low risk pregnancies, Norway 1989–2014.

**Fig 6:**
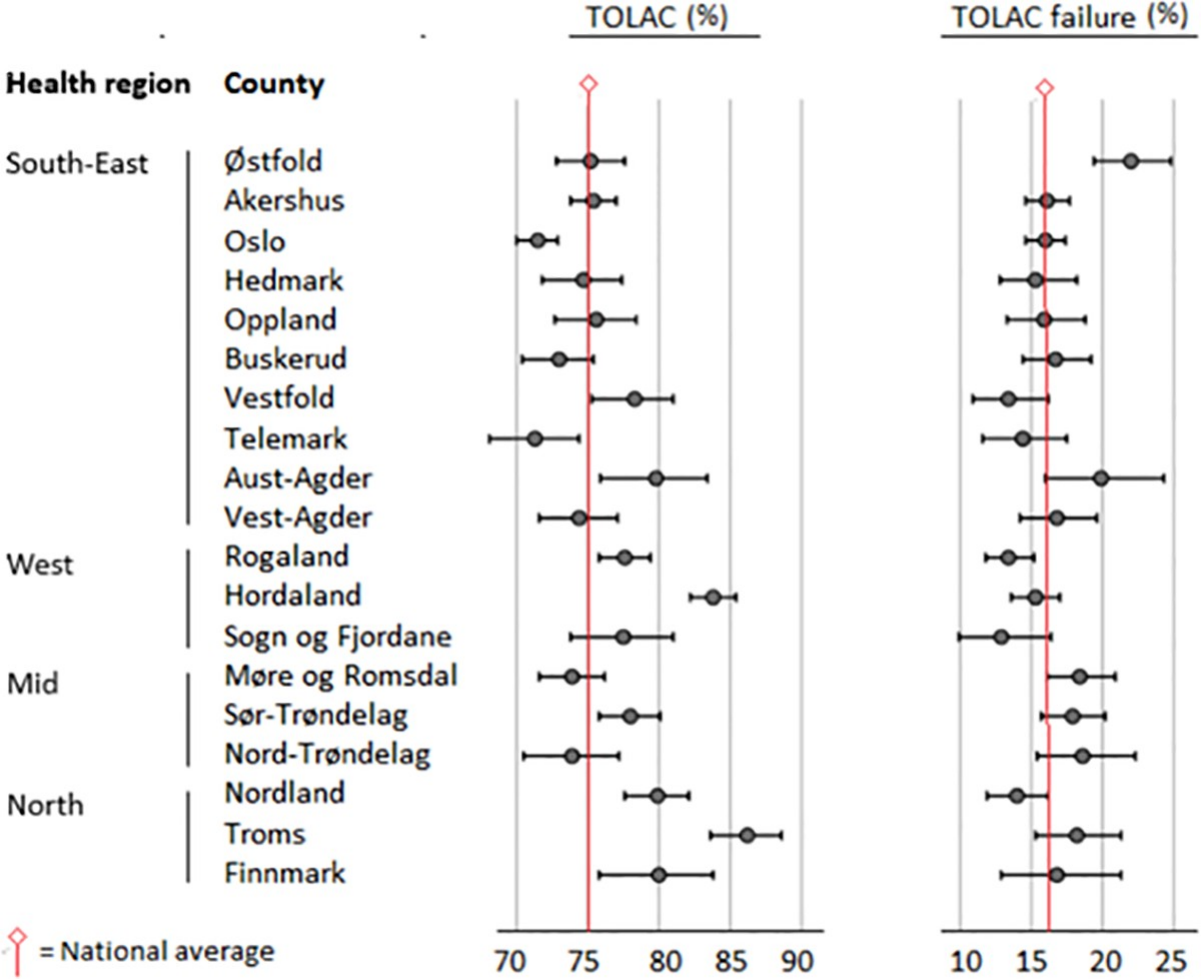
Trial of labor after cesarean section (TOLAC) and TOLAC failure rates (%) by county (95% CI), low risk deliveries, Norway 1989–2014.

In health region South East, the TOLAC rate was 72.2% (reference = 1), compared to 79.3% in health region West; RR 1.09 (1.07 to 1.11) ARR 1.06 (1.04 to 1.08), 73.9% in health region Mid; RR 1.02 (1.00 to 1.04) ARR 1.01 (0.99 to 1.03), and 80.6% in health region North; RR 1.11 (1.08 to 1.13) ARR 1.10 (1.08 to 1.12). In health region South East, the TOLAC failure rate was 16.6% (reference = 1), compared to 14.1%, in health region West; RR 0.86 (0.79 to 0.94) ARR 0.95 (0.96 to 1.05), 18.2% in health region Mid; RR 1.10 (1.01 to 1.21) ARR 1.21 (1.10 to 1.33) and 15.8% in health region North; RR 0.95 (0.86 to 1.07 ARR 1.02 (0.91 to 1.15). In the second half of the study period, a particular increase in TOLAC failure was seen in health region Mid (Fig 5). Significant rate differences, both in TOLAC and TOLAC failure, were seen between counties in the same health region (Fig 6).

## Discussion

Low TOLAC rates were observed in older mothers, and in women originating from East Asia or Latin America. High TOLAC failure rates were observed in older mothers, in women with short education, and in women of non-western origin. The effects of delivery unit size and region were considerable. Temporal changes were observed in the effects of several determinants: In the second half of the observation period, the association between non-TOLAC and high maternal age was weaker, the association between short education and TOLAC failure was stronger, and the association between TOLAC failure and small size of delivery unit was stronger.

The population-based design, a high number of births and use of validated outcome data represent strengths of the study. Several potential non-medical determinants were available and could be included in the analyses. However, whereas the size of the study population provided narrow CIs, some of the statistically significant effects observed were small (e.g. TOLAC and short education) and might be of limited practical consequence.

The low risk group was defined to select a study population with a minimum of residual medical risk, and linkage of first and second deliveries enabled exclusion of women with a record of recurrent medical indications. Still, some residual of unreported medical risk might persist. However, validations of data on medical conditions in the MBRN against hospital records has shown acceptable negative predictive values [12–17]. Thus, it does not appear likely this would be a cause of material confounding.

Different definitions of TOLAC and TOLAC failure hamper comparison of rates between studies. Particularly, all acute CS cases cannot be assumed to represent TOLAC and TOLAC failure. In this study, TOLAC was identified by using MBRN data that indicated an intention or attempt to deliver vaginally [10]. This reduced the number of unclassifiable cases and the risk of including acute CS non-TOLAC deliveries in the TOLAC group. Some misclassification of outcome cannot be ruled out but would in case most likely be non-differential.

In the present study, the observed TOLAC rate was higher, and the TOLAC failure rate was lower than in low risk pregnancies in other developed countries. In a Danish single-center study of TOLAC, in a setting of minimal risk, the TOLAC rate was 65% with a TOLAC failure rate of 33% [18]. In a low risk US population, New York state, 1998–2002, the TOLAC rate declined from 58.7% to 35.7% [19]. In Canada, a TOLAC rate of 21% has been reported in low risk pregnancies [20].

The observations of a lower TOLAC rates in older women in the present study contrast a widely cited US study of TOLAC complications, in which the opposite was reported [21]. In another observational US study of TOLAC outcome, there no age differences were reported between the TOLAC and non-TOLAC group [22]. However, our results agree well with more recent studies in the US and the UK [3, 23, 24].

High maternal age might cause biological effects, e.g. reduced tissue elasticity, and increased risk of ineffective labor. The partially time dependent effect of maternal age on TOLAC (Fig 2) is suggestive of changes in the perception of risk. As deliveries in older women have become more common, high maternal age might have been regarded as less of a contra indication to a vaginal delivery. However, the excess TOLAC failure rate observed in older mothers agrees well with previous research [3], and this effect did not significantly change with time.

The association observed between TOLAC and high education differs from results from studies of socioeconomic status in the US, UK and Hong Kong [24–26]. The observed association in the present study between low socioeconomic status and TOLAC failure is in line with results in the US, but not in the UK [24, 27]. The stronger association observed between TOLAC failure and short education in 2003–2014 compared to 1989–2002 could reflect generally increasing education levels, rendering women with short education relatively more disadvantaged.

In the US and UK, TOLAC has been associated with non-white ethnicity [3, 24, 28]. A study of CS in migrant women in Norway found high rates of planned CS in women from Latina America, and high rates of acute CS in women from Africa south of the Sahara, which agrees well with our observations [29]. A connection to CS rates in the woman’s country of origin has been reported [30]. This might explain the low TOLAC rate observed in women from Latin America but is contrasted by high TOLAC rates in North American women compared to rates in the US and Canada [19, 20, 31]. The excess TOLAC failure risk observed in non-western women, is in line with previous studies in the US, UK, and Sweden [3, 5, 24].

Some of the effects observed from socioeconomic status and maternal origin might be connected to a residual of unrecorded risk, or factors for which we could not fully account, e.g. a sedentary lifestyle [32–34]. However, the study concerned a low risk group, and it seems unlikely that women from all non-western regions should have undiagnosed and unrecorded medical risks affecting TOLAC failure to the extent observed. A more plausible explanation might be cultural and language barriers affecting use of antenatal care, the patient-doctor rapport, and clinical decisions [35–37].

Our findings indicate that a residual of inequality might persist in clinical management of TOLAC, even in a health system as in Norway, with free and available maternity care. The causal mechanisms are probably multiple, e.g. maternal attitudes, lifestyle factors, and practitioners’ attitudes. To explore this further and counter it on a policy level appears more appropriate than accepting the observed determinants as non-modifiable elements in clinical decision-making [35, 38].

Regarding the effect of delivery unit volume on TOLAC and TOLAC failure, studies are scant and comparison is hampered by differences in patient risk status [3]. However, it seems likely that low volume might reduce confidence and proficiency in clinical management of TOLAC. With 1000 yearly deliveries, presuming that national TOLAC rates apply, there would be approximately 40–50 TOLACs per year. This might be enough to maintain competence, but some important medical conditions would be rare.

In small and mid-size delivery units in Norway, a staff of 3–8 consultants cover shifts in the delivery unit, outpatient clinics in gynecology and obstetrics, as well as gynecological operations [39]. It appears likely that selection for TOLAC as the CS threshold in small and mid-sized hospitals could also be affected by organizational factors, e.g. capacity limitations [40]. Operation room availability, ease of transfer from the delivery room to the operating table, and available pediatric services are also be factors that might affect the appropriateness of attempting and continuing a TOLAC.

Even without national guidelines regarding TOLAC, the observed inter-regional ranges, 72% - 80% in TOLAC and 14% - 18% in TOLAC failure, were low compared to ranges reported in other studies, e.g. in Italy, Australia, and Germany [41–44]. Regional variation could reflect differences in attitude to the safety of the procedure. However, it could also represent necessary adaptions of evidence-based practice to local conditions, e.g. available resources, travel distances, and staffing. Still, close surveillance appears warranted, in particular concerning health-related maternal and offspring outcomes.

Interestingly, the observed effect of geographic region was on the same level as individual determinants considered relevant to planning of mode of delivery in previous research [5, 6]. Regarding TOLAC failure, for example, the effect of delivering in health region Mid vs region West, was higher than being 30 to 35 years vs under 25 years. Thus, assessment of individual TOLAC failure risk appears incomplete if delivery unit characteristics are left out of the equation.

## Conclusion

Low maternal age, high education, and western country of origin were associated with high TOLAC rates, and low TOLAC failure rates. Maternity unit characteristics (size and region) contributed with effects on the same level as individual determinants studied. Temporal changes were observed in determinant effects.
